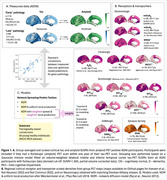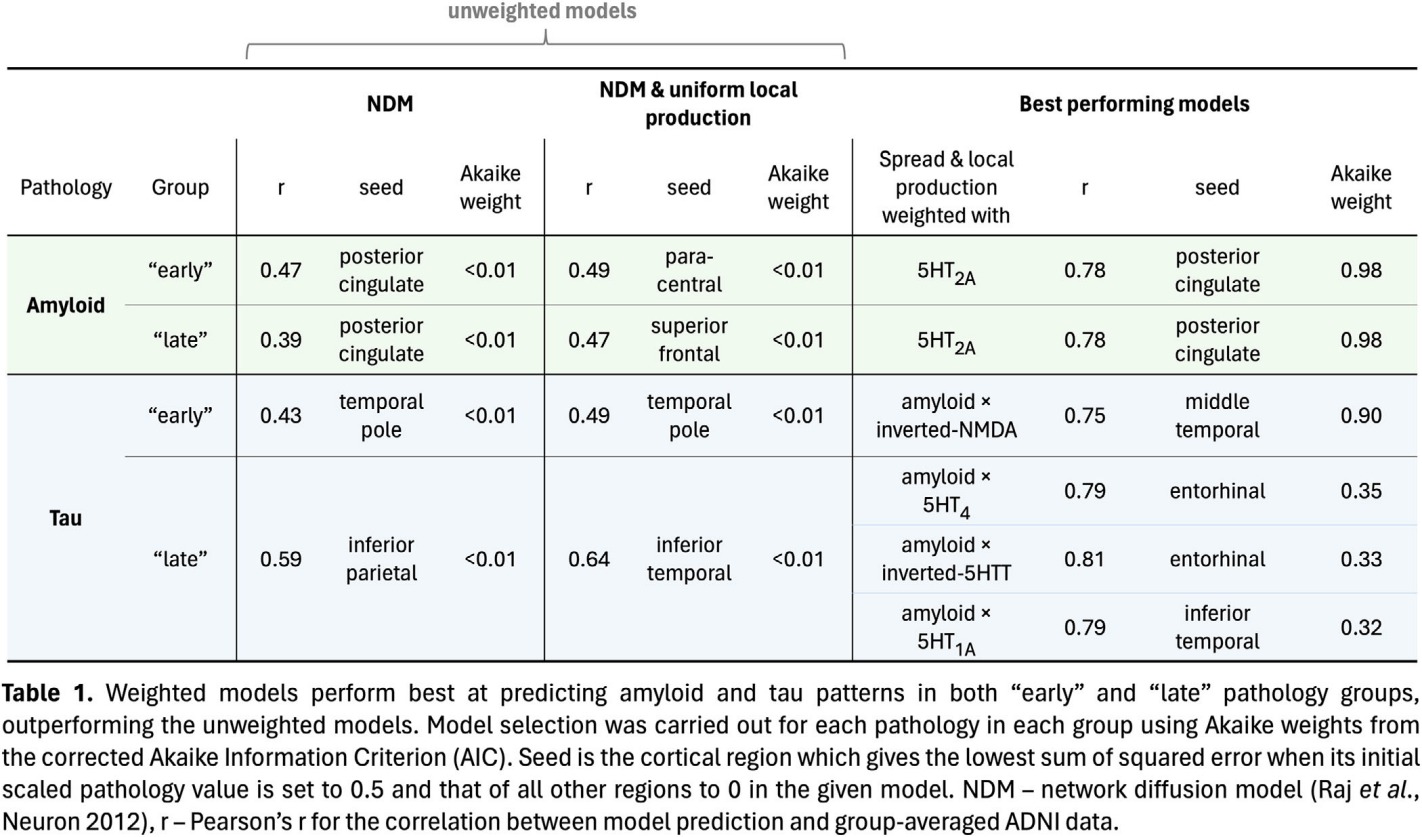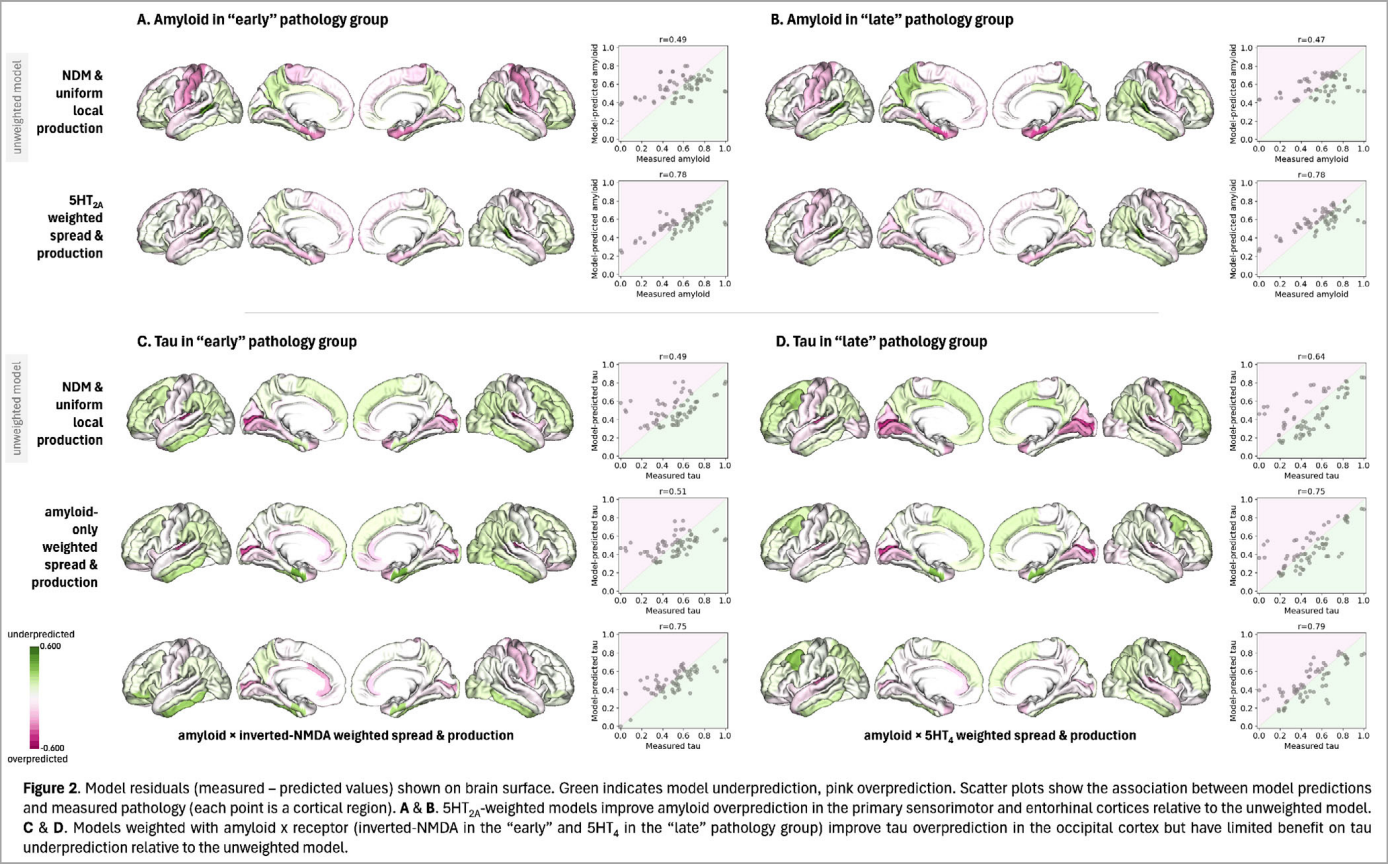# Effects of regional neurotransmitter receptor densities on modelling amyloid and tau accumulation in Alzheimer’s disease with network spreading models

**DOI:** 10.1002/alz.095454

**Published:** 2025-01-09

**Authors:** Sonja Soskic, Elinor Thompson, Tiantian He, Anna Schroder, Neil P Oxtoby, Daniel C Alexander

**Affiliations:** ^1^ Centre for Medical Image Computing, Department of Medical Physics and Biomedical Engineering, University College London, London United Kingdom; ^2^ Centre for Medical Image Computing, Department of Computer Science, University College London, London United Kingdom

## Abstract

**Background:**

Neurotransmitter receptors’ contribution to Alzheimer’s disease (AD) pathology development has been implicated by basic science studies but is yet to be fully established. Here, we incorporate receptor density maps into network spreading models to predict amyloid and tau patterns in AD, reflecting their potential roles in facilitating or impeding pathology production and connectivity‐mediated spread.

**Method:**

Amyloid‐PET positive individuals from the Alzheimer’s Disease Neuroimaging Initiative (ADNI) were divided into “early” (n = 119) and “late” (n = 69) pathology groups according to tau accumulation in the temporal cortex (Figure 1A). Cortical amyloid and tau SUVRs were group‐averaged and compared with model predictions using Pearson’s correlations.

Relevant regional cortical receptor densities (“receptors”) were obtained from PET data of healthy individuals (Hansen *et al.*, Nat Neurosci 2022) (Figure 1B).

We used our centre’s Network Spreading Models Toolbox (https://github.com/ucl‐pond/network_spreading_models) to model connectivity‐mediated spread of amyloid and tau. We use the receptors to weight the spread and additional local production model terms, and assess the advantage of these receptor‐weighted models in predicting pathology patterns over models without receptors (Figure 1C).

Receptors showing significant negative regional correlation with pathology at baseline, indicating a possible protective role, were inverted and scaled to range 0‐1 to downweigh model terms; otherwise, they were scaled to range 1‐2 to upweight the terms. For tau prediction, we also weighted model terms with group‐matched regional amyloid, and with the regional product of amyloid and receptors to reflect their interaction. Best performing models were selected using Akaike weights.

**Result:**

Amyloid deposition in both groups was modelled best by 5HT_2A_‐weighted models (r = 0.78) (Table 1, Figure 2A&B).

“Early” tau accumulation was best predicted by weighting with amyloid x inverted‐NMDA (r = 0.75), outperforming amyloid‐only weighting (r = 0.51) (Figure 2C).

For “late” tau accumulation, top three models performed similarly: weighting with amyloid x 5HT_4_ (r = 0.79), x inverted‐5HTT (r = 0.81) or x 5HT_1A_ (r = 0.79). Amyloid‐only weighting also performed well (r = 0.75) (Figure 2D), and better than weighting with any receptor alone.

**Conclusion:**

Neurotransmitter receptor densities can improve model predictions of amyloid and tau patterns in the AD continuum. Serotonergic and glutamatergic receptors appear most predictive, implying their possible role in AD pathology progression.